# MEBANet: A Multi-domain Enhancement and Boundary Awareness Network for urban village extraction from high-resolution imagery

**DOI:** 10.1371/journal.pone.0330302

**Published:** 2025-10-22

**Authors:** Fangzhe Chang, Xiaoyong Fan, Ruining Xu, Shuhai Wang, Kun Qin, Xuming Gao

**Affiliations:** 1 Hebei Provincial Communications Planning, Design, and Research Institute Co., Ltd, Shijiazhuang, China; 2 College of Geoscience and Surveying Engineering, China University of Mining and Technology (Beijing), Beijing, China; 3 Hebei Transportation Investment Group Company Limited, Shijiazhuang, China; 4 School of Information Science and Technology, Shijiazhuang Tiedao University, Shijiazhuang, China; South China University of Technology, CHINA

## Abstract

Urban villages, as a typical phenomenon in the process of urbanization, play a significant role in urban planning and sustainable development. However, their high-density structures and complex boundaries pose significant challenges for extraction tasks based on remote sensing imagery. To address these challenges, this paper proposes a Multi-domain Enhancement and Boundary Awareness Network (MEBANet) for urban village extraction. MEBANet consists of three core blocks: 1) The spatial-frequency-channel feature extraction block (SFCB), which simultaneously enhances feature representation in the spatial, frequency, and channel domains; 2) The multi-scale boundary awareness block (MBAB), which leverages dense atrous spatial pyramid pooling (DenseASPP) and multi-directional sobel operator convolution to strengthen the perception of complex boundaries; and 3) The deep supervision block (DSB), which accelerates model convergence through multi-level supervision signals. Experiments were conducted on three publicly available datasets from Beijing, Xi’an, and Shenzhen. The results demonstrate that MEBANet outperforms existing methods in terms of precision, recall, F1-score, and IoU. Additionally, cross-dataset transfer experiments validate the robustness and generalization capability of MEBANet. Ablation studies further confirm the effectiveness of each block. This study provides a high-accuracy and automated solution for urban village extraction from high-resolution remote sensing imagery, offering valuable insights for urban planning and management.

## Introduction

Urban villages typically refer to residential settlements originally of rural nature that, despite being located within urban areas, have not been fully integrated into formal urban governance and infrastructure systems during the process of urbanization [1,2]. With the rapid advancement of urbanization, urban villages have emerged as a unique socio-spatial phenomenon at the intersection of rural and urban development [3]. Characterized by high-density, small-scale buildings, urban villages accommodate a significant portion of the urban population and resources, providing affordable housing for low-income residents [4]. However, the negative impacts associated with urban villages cannot be overlooked. These areas often pose serious social, economic, and environmental challenges. For instance, their densely packed and disorderly layout disrupts the urban landscape; inadequate infrastructure lowers residents’ quality of life; and poor sanitation conditions, coupled with limited public services, hinder sustainable urban development [5,6]. Therefore, accurately delineating the spatial extent of urban villages is not only a critical task for urban planning and governance but also a fundamental prerequisite for achieving refined urban management and sustainable development.

Traditional methods for mapping the spatial extent of urban villages primarily rely on field surveys [4,7]. Although these methods yield reliable results, they are constrained by high costs, intensive labor requirements, and long execution periods, factors that limit their scalability when applied to widely distributed urban villages [8]. With the rapid advancement of remote sensing technology, high-resolution imagery has emerged as a promising alternative for urban village extraction. Remote sensing imagery offers wide spatial coverage and frequent updates, making it an increasingly important data source for extracting urban village spatial information [1,9]. However, most Traditional approaches still depend on visual interpretation. While this avoids the complexities of field surveys, visual interpretation remains limited by inconsistent interpretation standards and delayed data updates, thus failing to meet the demands of large-scale and efficient urban village monitoring [4]. Consequently, there is an urgent need for automated solutions.

Currently, mainstream approaches for automated feature extraction can be broadly categorized into traditional machine learning methods and deep learning methods [10]. Traditional machine learning algorithms, such as Support Vector Machines (SVM) and Random Forests (RF), are known for their computational simplicity, fast processing speed, and relatively low data requirements [11]. These methods have been widely applied in urban village extraction tasks. For example, Duque et al. [5] evaluated the performance of logistic regression, SVM, and RF in cities such as Buenos Aires (Argentina), Medellín (Colombia), and Recife (Brazil), demonstrating that SVM achieved the best results. Matarira et al. [1] successfully delineated urban villages in Durban, South Africa, by combining the random forest algorithm with Sentinel-2A imagery. Similarly, Park et al. [12] mapped the spatial distribution of urban villages in Ulaanbaatar from 1990 to 2013 using QuickBird and Landsat images supported by SVM. Gevaert et al. [6] further applied SVM to extract urban villages in Kigali, Rwanda, and Maldonado, Uruguay. Nevertheless, these traditional machine learning methods heavily rely on handcrafted features, which are often incomplete and prone to overfitting. Moreover, they struggle to model complex non-linear relationships in heterogeneous environments, ultimately limiting classification accuracy [13]. With the rise of deep learning, significant advancements have been made in this domain. Deep learning constructs robust nonlinear neural networks capable of automatically learning hierarchical feature representations, eliminating the need for manual feature engineering [7,9,14]. As a result, an increasing number of deep neural network-based models have been employed in extraction tasks for identifying objects of interest in remote sensing imagery, achieving remarkable performance. This includes widely used classical semantic segmentation networks such as FCN [15], U-Net [16], and DeepLabv3+ [17], as well as specialized models designed for specific land cover types or challenges. For instance, Wang et al. [18] proposed RFENet and addressed the vulnerability of deep learning models in aerial image segmentation to adversarial patch attacks. Zhang et al. [19] introduced C_ASegformer to improve multi-scale feature integration and contextual awareness, achieving superior results compared to existing models. In the context of urban village extraction, several deep learning-based studies have demonstrated promising results. Verma et al. [2] proposed the use of pre-trained convolutional networks for detecting urban village in Mumbai. Persello et al. [20] applied FCN to extract urban villages in Dar es Salaam, Tanzania. Similarly, Pan et al. [4] achieved delineation of urban villages in Guangzhou, China, using a U-Net-based method. Wei et al. [7] compared the performance of FCN, U-Net, and ResUNet for urban village extraction at the junction of Dongguan, Huizhou, and Guangzhou. These studies primarily rely on the direct application of existing semantic segmentation networks without considering the unique characteristics of urban villages, thereby limiting further improvements in accuracy.

To address these limitations, some researchers have proposed tailored architectures. For example, Ansari et al. developed a composite model that integrates U-Net with a multiscale contourlet transform to improve extraction accuracy in the cities of Mumbai and Pune, India. Fan et al. [13] introduced UisNet, which enhances extraction accuracy by utilizing building-level information. Based on a Transformer architecture, UisNet incorporates multimodal data including spatial building footprints and floor numbers to extract urban villages in Shenzhen. Du et al. [21] proposed STMNet, which fuses texture features derived from gray-level co-occurrence matrices with original images to extract urban villages in Beijing. Zhang et al. [22] introduced UV-SAM to address inaccurate boundary extraction by incorporating a prompt generation module into a foundational model, achieving promising results in Beijing and Xi’an. Furthermore, Li et al. [23] addressed the limited global context modeling of CNNs and the computational complexity of Transformers by introducing the Mamba architecture for the first time in this task, resulting in the UV-Mamba.

Despite recent advancements, most deep learning-based methods for urban village extraction rely primarily on spatial-domain representations, with limited exploration of the frequency domain. Spatial-domain approaches primarily operate on raw pixel intensities and spatial arrangements, which may not fully reveal the underlying structural patterns, especially in highly heterogeneous and visually complex urban scenes [24,25]. In contrast, frequency-domain representations offer a complementary perspective by decomposing images into components with distinct frequency characteristics. Low-frequency components capture global structures and smooth variations, while high-frequency components emphasize fine-grained textures, edges, and discontinuities-features that are often critical for delineating urban villages with irregular layouts and noisy visual characteristics. Integrating frequency-domain information enables models to enhance their perception of subtle yet important features that may be overlooked in the spatial domain, thereby improving the overall robustness and precision of segmentation [26]. As such, a combined spatial-frequency perspective allows for richer and more discriminative feature representation, which is particularly beneficial in complex urban extraction tasks.

Encouragingly, recent studies have attempted to incorporate frequency-domain information into semantic segmentation tasks. Existing approaches can be broadly categorized into two strategies. The first involves parallel use of frequency-domain and spatial-domain feature extraction modules, often implemented as dual-branch networks or as separate modules inserted into the encoder and decoder stages, respectively [27,28]. The second approach introduces frequency-domain features as auxiliary enhancement modules to support spatial feature learning [29,30]. However, the former design increases computational cost and limits timely feature interaction, while the latter cannot fully exploit frequency information.

To address the aforementioned challenges, this study proposes a novel method named multi-domain enhancement and boundary awareness network (MEBANet). MEBANet is primarily composed of three core modules. The spatial-frequency-channel feature extraction block (SFCB) enhances multi-domain feature representations, particularly by incorporating frequency-domain processing to effectively extract critical yet often overlooked features. The multi-scale boundary awareness block (MBAB) leverages multi-scale feature extraction and boundary awareness to improve the accuracy of boundary delineation. Finally, a deep supervision block (DSB) is introduced to accelerate network convergence. The main contributions of this study can be summarized as follows:

1) We propose a novel MEBANet architecture that integrates multi-domain feature enhancement and multi-scale boundary awareness mechanisms to capture the complex spatial characteristics of urban villages more accurately. Its effectiveness is demonstrated through experiments on datasets from Beijing, Xi’an, and Shenzhen.
2) We design the SFCB module to simultaneously enhance features in the spatial, frequency, and channel domains. Unlike existing studies, our approach fuses spatial-domain enhancement submodule, frequency-domain enhancement submodule, and channel-domain enhancement submodule into a unified block that enables comprehensive feature interaction during extraction. Moreover, the design allows for flexible stage-wise integration within the network. Experimental results validate the effectiveness of SFCB in enhancing representational capacity.
3) We propose the MBAB module, which integrates DenseASPP and Sobel convolution to enhance boundary awareness. DenseASPP captures multi-scale features to address the spatial heterogeneity and scale variation of urban villages, while Sobel convolution refines boundary localization. This design significantly improves segmentation accuracy by reducing false positives and missed detections around complex boundaries.

## Proposed methods

### Overall structure

**Fig 1:**
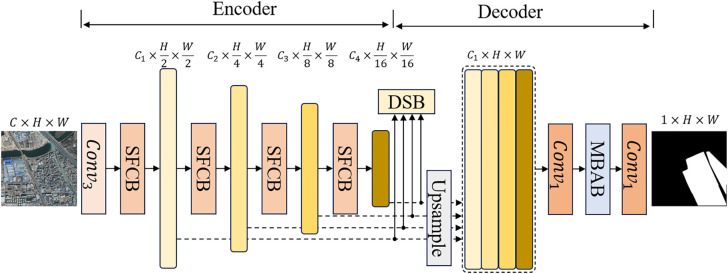
The structure of MEBANet.

The architecture of the proposed MEBANet is illustrated in Fig 1, which consists of an encoder and a decoder. In the encoder, a 3 × 3 convolution is first applied for initial feature extraction. Subsequently, the proposed SFCB is employed as the backbone to hierarchically extract multi-level features. The encoder comprises four SFCB, each consisting of a frequency-domain enhancement submodule (FDE), a spatial-domain enhancement submodule (SDE), and a channel-domain enhancement submodule (CDE). These extractors output four levels of features, denoted as $f_{e\text{1}}$, $f_{e\text{2}}$, $f_{e\text{3}}$, $f_{e\text{4}}$. Assuming the input image has a size of (3,H,W), the output feature from the encoder are of dimensions ($C_{\text{1}},H/\text{2},W/\text{2}$), ($C_{\text{2}},H/\text{4},W/\text{4}$), ($C_{\text{3}},H/\text{8},W/\text{8}$), and ($C_{\text{4}},H/\text{1}\text{6},W/\text{1}\text{6}$), where $C_{i}=\text{3}\text{2}\times i$, and i∈{1,2,3,4}.

In the decoder, instead of adopting a complex decoding structure, all features from the encoder are upsampled to match the input resolution. Each upsampled feature map is adjusted to have a uniform channel dimension of $C_{\text{1}}$, and then concatenated to form a unified feature representation of size $\left({\text{4}\times C}_{\text{1}},H,W\right)$. To enhance the model’s adaptability to the shape and scale variations of urban villages, the MBAB is incorporated before generating the final output. Finally, the DSB is introduced to accelerate model convergence and enhance training efficiency. It leverages the intermediate features $f_{e\text{1}}$, $f_{e\text{2}}$, $f_{e\text{3}}$, $f_{e\text{4}}$ from the encoder, combined with the output of the MBAB and the result of a 1 × 1 convolution, to provide auxiliary supervision signals that facilitate model optimization [31]. The subsequent sections of this chapter will provide a detailed explanation of the design and implementation of the SFCB, MBAB, and DSB.

### Feature extraction using SFCB

**Fig 2:**
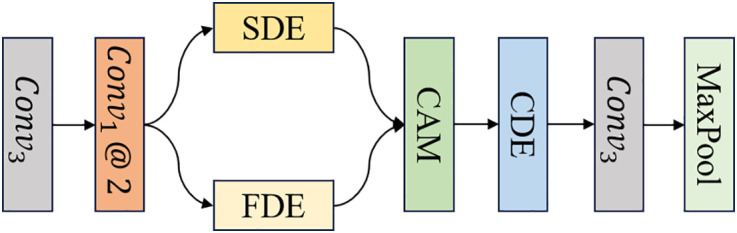
The structure of SFCB.


SFCB: The design of the SFCB aims to achieve efficient feature extraction and representation through multi-dimensional enhancement across the frequency, spatial, and spectral domains. As illustrated in Fig 2, the input feature first passes through a 3 × 3 convolution for initial processing. Then, two 1 × 1 convolutions are applied to generate two feature, which are respectively fed into the FDE and SDE. The use of 1 × 1 convolutions serve to reduce the number of channels, thereby improving computational efficiency, while also enabling the separation of features that are more suitable for spatial-domain and frequency-domain enhancement. As a result, two distinct feature maps are obtained: $F_{\text{1}}$, which is passed to the SDE, and $F_{\text{2}}$, which is passed to the FDE.

The SDE outputs a refined feature map $F_{S}$, while the FDE produces $F_{F}$. These two outputs are fused through a cross-domain integration mechanism (CAM) to generate $F_{SF}$, which is then passed into the CDE. This module recalibrates the importance of each feature channel, resulting in the output feature $F_{C}$. Finally, a 3 × 3 convolution followed by a max-pooling operation is applied to downsample the features, producing the SFCB output $f_{ei}$ where i∈{1,2,3,4}. This process can be summarized as follows:

$$F_{\text{1}},F_{\text{2}}={\text{2}@Conv}_{\text{1}}({Conv}_{\text{3}}(F_{in}))$$ (1)

$$F_{S},F_{F}=SDE\left(F_{\text{1}}\right),FDE(F_{\text{2}})$$ (2)

$$F_{SF}=CAM(F_{S},F_{F})$$ (3)

$$F_{C}=CDE(F_{SF})$$ (4)

$$f_{ei}=MaxPool({Conv}_{\text{3}}\left(F_{C}\right))$$ (5)

where ${Conv}_{\text{3}}(\cdot )$,$\;{Conv}_{\text{1}}(\cdot )$ and MaxPool(·denote the 3 × 3 convolution, 1 × 1 convolution, and max-pooling operations, respectively. SDE(·), FDE(·), CAM(·) and CDE(·) represent the SDE, FDE, CAM, and CDE, respectively.

**Fig 3:**
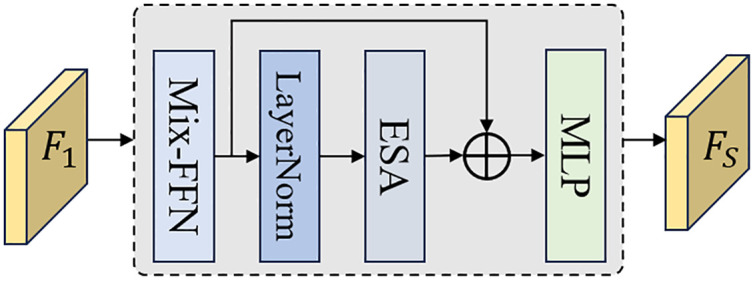
The structure of SDE.


SDE: The structure of the SDE is illustrated in Fig 3. Its core lies in leveraging the Transformer module to extract global spatial information. To enhance computational efficiency, we adopted a combination of the Mix-FFN and Efficient Self-Attention mechanism (ESA) [32]. The Mix-FFN automatically computes positional encodings from the input features using 3 × 3 convolutions, eliminating the complexity of adding positional encodings separately, as in Vision Transformer [33]. This design not only preserves positional information but also significantly improves computational efficiency. This process can be described as follows:

$$F_{out}=MLP\left(Gelu\left({Conv}_{\text{3}}\left(F_{in}\right)\right)\right)+F_{in}$$ (6)

where, $F_{in}$ and $F_{out}$ represent the input and output features of the Mix-FFN, respectively, Gelu(·denotes the GELU activation function, and MLP(·) refers to the multi-layer perceptron.

The ESA differs from Vision Transformer in that it introduces a parameter R to compress the key and value matrices, significantly reducing the computational cost of multi-head attention. First, the query (Q), key (K), and value (V) are computed, a process that can be defined as follows:


$$Q,K,V={MLP}_{Q}\left(F_{in}\right),{MLP}_{K}\left(F_{in}\right),{MLP}_{V}\left(F_{in}\right)$$ (7)

where, ${MLP}_{Q}$, ${MLP}_{K}$, and ${MLP}_{V}$ represent the multi-layer perceptron used to compute Q, K, and V, respectively. Typically, Q, K, and V share the same dimensions (B,head,N,C), where B denotes the batch size, head represents the number of attention heads, N is the sequence length, and C is the embedding dimension per head. The introduction of R first reshapes K and V into (B,head,N/R,C×R), and then uses a multi-layer perceptron to transform K and V into the size (B,head,N/R,C). This process can be described as follows:


$$\left(\overset{―}{K},\overset{―}{V})=Reshape(N/R,C\times R)(K,V\right)$$ (8)

$$K,V={MLP}_{C\times R\to C}(\overset{―}{K},\overset{―}{V})$$ (9)

The output features $F_{out}$ of the ESA can be described as:


$$F_{out}=(\sigma (Q\times K^{T})/\sqrt{d_{K^{T}}})\;\times V$$ (10)

**Fig 4:**
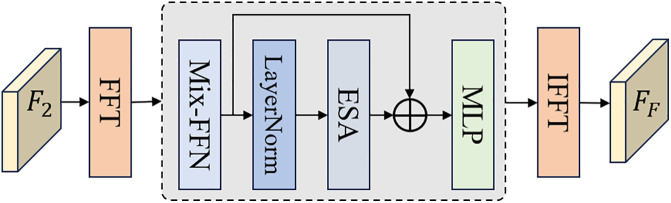
The structure of FDE.

FDE: The structure of the FDE is illustrated in Fig 4. The primary distinction from the SDE lies in the use of the Fast Fourier Transform (FFT) to map the input features from the spatial domain to the frequency domain before feeding them into the Transformer module. In the frequency domain, the features are adjusted and then remapped back to the spatial domain via the Inverse Fast Fourier Transform (IFFT) [34]. This process effectively captures frequency information that is difficult to extract in the spatial domain.

**Fig 5:**
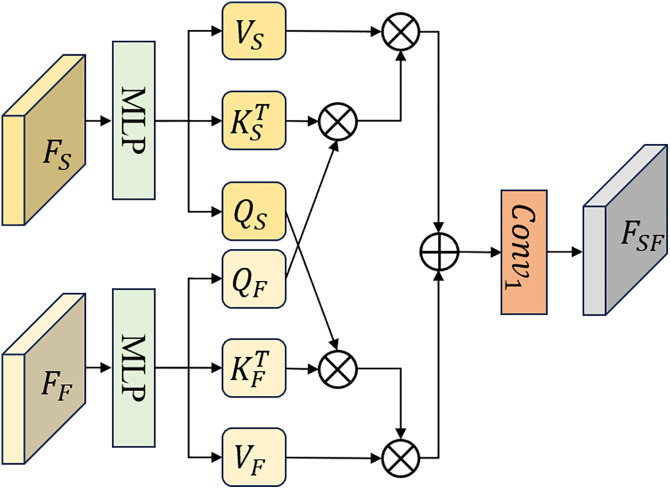
The structure of CAM.


CAM: The structure of the CAM is illustrated in Fig 5. CAM integrates the outputs of the FDE and SDE through cross attention mechanism [35]. This process can be described as follows:

$$Q_{S},K_{S},V_{S}={MLP}_{Q}\left(F_{S}\right),{MLP}_{K}\left(F_{S}\right),{MLP}_{V}(F_{S})$$ (11)

$$Q_{F},K_{F},V_{F}={MLP}_{Q}\left(F_{F}\right),{MLP}_{K}\left(F_{F}\right),{MLP}_{V}(F_{F})$$ (12)

$$A_{F}={\sigma (Q}_{S}\times K_{F}/\sqrt{d_{K_{F}}})$$ (13)

$$A_{S}={\sigma (Q}_{F}\times K_{S}/\sqrt{d_{K_{S}}})$$ (14)

$$F_{SF}=A_{F}\times V_{F}+A_{S}\times V_{S}$$ (15)

Where σ(·) represents the softmax activation function, and $d_{K}$ is the channel dimension of K.

**Fig 6:**
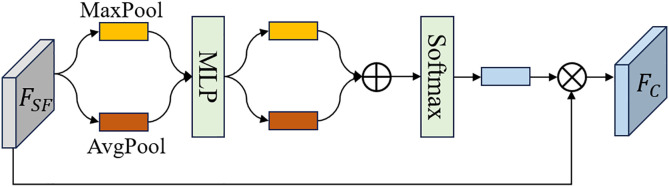
The structure of CDE.


CDE: The structure of the CDE is illustrated in Fig 6. The input features are first processed through max-pooling and average-pooling, followed by MLP and softmax to compute the importance weights for each channel. These weights are then multiplied with the original input features to produce the output feature $F_{C}$ [36]. This process can be described as follows:

$$F_{C}={Conv}_{\text{3}}(\sigma \left(MLP\left(AvgPool\left(F_{SF}\right)\right)+MLP\left(MaxPool\left(F_{SF}\right)\right)\right){\times F}_{SF})$$ (16)

Where AvgPool(·) denotes the average-pooling operation, and MaxPool(·) represents the max-pooling operation. Finally, the channel-enhanced feature $F_{C}$ is processed through a 3 × 3 convolutional layer followed by a max pooling operation, generating the output feature $F_{ei}$ of the SFCB.

### MBAB for enhancing boundary information

**Fig 7:**
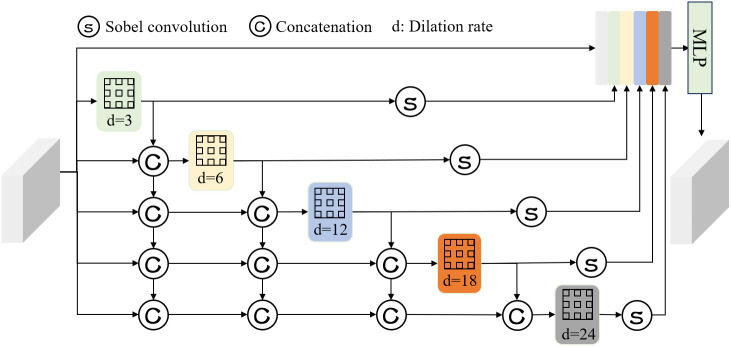
The structure of MBAB.

The primary objective of the MBAB is to enhance the model’s adaptability to the shape and scale variations of urban villages while strengthening the extraction of boundary details. The structure of this module is illustrated in Fig 7, and its core consists of two components: dense atrous spatial pyramid pooling (DenseASPP) [37] and sobel operator convolution [38].

The DenseASPP comprises six parallel branches. One branch employs a residual connection, directly passing the input features without any processing, while the other five branches utilize atrous convolutions with dilation rates of 3, 6, 12, 18, and 24, respectively. This multi-scale design captures semantic information at different receptive fields, enabling adaptation to the complex shape and scale variations in urban village regions. Additionally, dense connections between the branches facilitate top-down multi-scale feature aggregation, ensuring that the output of each branch not only retains semantic information at the current scale but also integrates features from adjacent branches, significantly enhancing the overall feature representation capability. To further improve the extraction of boundary details, we introduce multi-directional Sobel operator convolution after each atrous convolution branch. Specifically, the input features are processed using eight directional Sobel convolution kernels, and the results from all directions are summed to produce the final feature. The eight directional Sobel kernels are defined as follows: $K_{\text{0}}=\{(\text{1},\text{2},\text{1}),(\text{0},\text{0},\text{0}),(-\text{1},-\text{2},-\text{1}\}$, $K_{\text{4}\text{5}}=\{(\text{2},\text{1},\text{0}),(\text{1},\text{0},-\text{1}),(\text{0},-\text{1},-\text{2}\}$, $K_{\text{9}\text{0}}=\{(\text{1},\text{0},-\text{1}),(\text{2},\text{0},-\text{2}),(\text{1},\text{0},-\text{1}\}$, $K_{\text{1}\text{3}\text{5}}=\{(\text{0},-\text{1},-\text{2}),(\text{1},\text{0},-\text{1}),(\text{2},\text{1},\text{0}\}$,$K_{\text{1}\text{8}\text{0}}=\{(-\text{1},-\text{2},-\text{1}),(\text{0},\text{0},\text{0}),(\text{1},\text{2},\text{1}\}$, $K_{\text{2}\text{2}\text{5}}=\{(-\text{2},-\text{1},\text{0}),(-\text{1},\text{0},\text{1}),(\text{0},\text{1},\text{2}\}$, $K_{\text{2}\text{7}\text{0}}=\{(-\text{1},\text{0},\text{1}),(-\text{2},\text{0},\text{2}),(-\text{1},\text{0},\text{1}\}$, $K_{\text{3}\text{1}\text{5}}=(\text{0},\text{1},\text{2}),(-\text{1},\text{0},\text{1}),(-\text{2},-\text{1},\text{0})$. The Sobel operator enhances edges in the feature map by computing pixel gradient changes, effectively improving the model’s sensitivity to boundary information [39]. This multi-directional convolution design not only captures edge features at different angles but also strengthens the model’s perception of complex boundary structures.

### DSB for network training

Deep Supervision is a training strategy for neural networks that not only utilizes the final output of urban village extraction to provide supervision signals but also incorporates intermediate outputs from different layers of the network to guide the training process. This strategy aims to address issues such as gradient vanishing and exploding in network training, while also improving the convergence speed and overall performance of the network [31,40].

We first unsampled the four features from the encoder, concatenate them, and then process them through the MBAB module and a 1 × 1 convolution to obtain the final output result$\;p_{final}$. This process can be described as:


$$p_{final}={Conv}_{\text{1}}(MBSB\left({Conv}_{\text{1}}\left(Cat(UP(f_{e\text{1}},f_{e\text{2}},f_{e\text{3}},f_{e\text{4}})\right)\right)))$$ (17)

where UP(·) denotes the upsampling operation, Cat(·) represents feature concatenation. Next, we utilize $f_{e\text{1}}$, $f_{e\text{2}}$, $f_{e\text{3}}$, and $f_{e\text{4}}$ along with the MBAB and 1 × 1 convolution to generate urban village extraction results at different scales as intermediate outputs of MEBANet. This process can be described as:


$$p_{i}={Conv}_{\text{1}}(MBSB\left(f_{ei}\right))$$ (18)

Finally, we denote the ground truth corresponding to $p_{final}$ as$\;y_{final}$, and apply max pooling operations to$\;y_{final}$ to obtain the ground truth for each additional output result. This process can be described as:


$$y_{i}=MaxPool\left(y_{final}\right),\;i\in \{\text{1},\text{2},\text{3},\text{4}\}$$ (19)

In this study, the loss for each component is calculated using the cross-entropy loss function [41], which can be defined as:


$$l=-\frac{\text{1}}{N}[ylog(p)+(\text{1}-y)log(\text{1}-p)]$$ (20)

where y represents the ground truth labels, p denotes the predicted probabilities from the model, and N is the total number of pixels. Therefore, the overall loss function for this study can be defined as:


$$Loss=l_{final}+{\beta \times l}_{intermediate}=l_{final}+\beta (l_{\text{1}}+l_{\text{2}}+l_{\text{3}}+l_{\text{4}})$$ (21)

where β is a balancing coefficient used to adjust the weight between the supervision of the final result and the intermediate results.

## Experiments and results

### Dataset and interpretation signs

Dataset: In this study, we conducted experimental validation using three datasets collected from Beijing, Xi’an, and Shenzhen. The remote sensing images include data from Beijing in 2016, Xi’an in 2018, and Shenzhen in 2020, with corresponding labels generated by professionals through visual interpretation and cross-validation methods. To fully leverage the dataset and evaluate the model’s generalization ability, we combined the training, testing, and validation sets and employed a 5-fold cross-validation approach to assess model accuracy [42]. During the experiments, the original images and their corresponding labels were cropped into non-overlapping 512 × 512-pixel patches to facilitate model training and testing. To enhance the diversity of the training data, we applied a series of image preprocessing methods for data augmentation, including:1) Image Rotation: Rotating the images and labels by 90°, 180°, and 270°; 2) Image Flipping: Horizontal flipping and vertical flipping; 3) Noise Addition: Gaussian noise, salt-and-pepper noise; 4) Image Blurring: Gaussian blur, mean blur, median blur [31].

**Fig 8:**
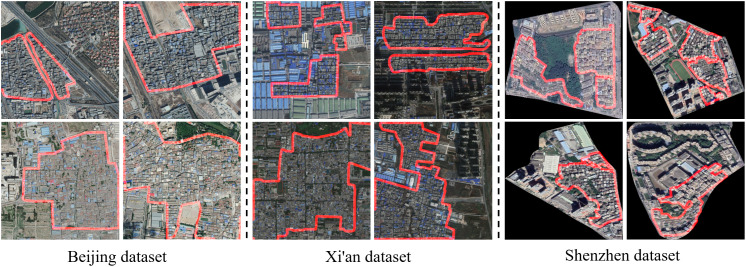
Example of an urban village. The boundary of the urban village is highlighted in red. The figure was produced by the authors of this study using the publicly available dataset from [13] and [22].

Interpretation signs: Due to the disorderly construction of urban villages, they exhibit distinct features in images, as shown in Fig 8. These features include: 1) Irregular Shapes: The lack of planning in the construction of urban villages results in irregular shapes, distinguishing them from commercial or well-planned residential areas. This leads to complex geometric forms in remote sensing imagery. 2) Internal objects: In urban villages, buildings are densely packed with narrow gaps between them, resulting in large, continuous, and compact rooftop-covered areas in the imagery. The individual buildings are typically small in scale, and the internal roads are often very narrow, winding, and lack proper planning. Additionally, public green spaces and centralized green areas are extremely scarce. 3) Rough Textures: The texture features are frequently changing and chaotic, with high spatial complexity, exhibiting strong contrast with the surrounding environment.

### Implementation details

The experiments in this study were conducted on a 64-bit Windows 10 operating system with an Intel(R) Core (TM) i9-11900K @ 3.50 GHz 16-core processor and an NVIDIA GeForce RTX 3090 GPU with 24 GB of memory. The programming language used was Python 3.8, with PyCharm as the development environment and PyTorch 2.0.0 as the deep learning framework.

During the training phase, manual tuning was employed to identify the optimal batch size, optimizer, learning rate, and β. The final batch size was set to 16, with AdamW selected as the optimizer. The learning rate was set to 1e-3, and β was set to 0.25. A cosine annealing strategy was applied to dynamically adjust it throughout training. The cross-entropy loss function was used for model optimization. To ensure that the loss curve stabilized, the maximum number of training epochs was set to 100.

To evaluate the model’s performance, we selected four commonly used metrics, including Precision (P), Recall (R), F1−score (F1), and Intersection over Union (IoU[43,44]. Additionally, we evaluated the model’s complexities by measuring floating point operations per second (FLOPs), parameters, and the inference time required for processing images of size 3 × 512 × 512.

To assess the performance of the proposed method, we compared it with three publicly available urban village extraction methods, namely UisNet [13] and UV-SAM [22], as well as three classic land cover classification methods, including ABCNet [45], CMTFNet [46], and UNetFormer [43]. To ensure the stability and reliability of the results, all experiments in this study are repeated five times and the results are averaged.

## Experimental results

**Fig 9:**
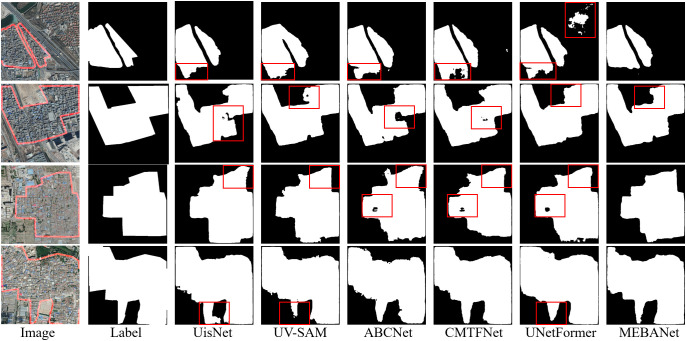
Urban village extraction results on the Beijing dataset. The figure was produced by the authors of this study using the publicly available dataset from [22].

**Fig 10:**
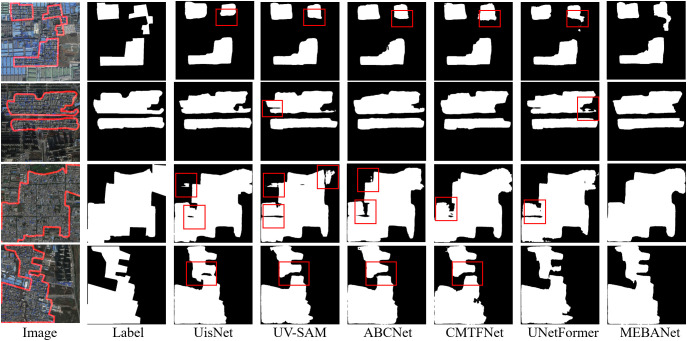
Urban village extraction results on the Xi’an dataset. The figure was produced by the authors of this study using the publicly available dataset from [22].

**Fig 11:**
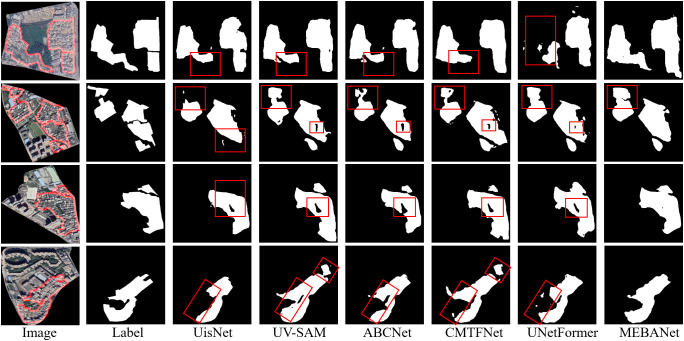
Urban village extraction results on the Shenzhen dataset. The figure was produced by the authors of this study using the publicly available dataset from [13].

Qualitative analysis: Figs 9–11 present the extraction results of six method on the Beijing, Xi’an, and Shenzhen datasets, respectively. From the figures, it is evident that urban villages exhibit significant heterogeneity in shape and scale, posing considerable challenges for accurate identification. Among the compared methods, except for MEBANet, the other methods generally suffer from issues such as false positives, false negatives, irregular boundaries, and internal holes. In contrast, MEBANet demonstrates superior performance in its extraction results. It not only captures the complete contours of urban villages with clear and continuous boundaries but also achieves the highest alignment with the ground truth labels, with almost no noticeable false positives or false negatives. Furthermore, MEBANet exhibits strong robustness in handling the complex internal structures and scale variations of urban villages, effectively reducing internal voids. These results further validate the superiority of MEBANet in urban village extraction tasks.

Quantitative Evaluation: Table 1 presents the quantitative evaluation results of six methods on the Beijing dataset. It can be observed that the proposed MEBANet achieves the highest performance across all evaluation metrics, with P of 89.28%, R of 88.47%, F1 of 88.87%, and IoU of 79.97%. Among the remaining methods, UisNet ranks second, achieving 86.74% in P, 87.96% in R, 87.35% in F1, and 77.53% in IoU. In contrast, UNetFormer performs the worst in three of the four metrics, namely P, F1, and IoU, while CMTFNet records the lowest R at 85.59%. Table 2 summarizes the quantitative evaluation results on the Xi’an dataset. MEBANet again demonstrates superior performance, with P of 92.78%, R of 89.89%, F1 of 91.31%, and IoU of 84.01%. UisNet ranks second, achieving 90.63% in P, 89.39% in R, 90.01% in F1, and 81.83% in IoU. ABCNet performs the worst across all metrics, with scores of 89.03%, 86.83%, 87.92%, and 78.44%, respectively. Table 3 reports the evaluation results on the Shenzhen dataset. MEBANet consistently achieves the best performance, with P of 88.44%, R of 87.29%, F1 of 87.86%, and IoU of 78.35%. CMTFNet ranks second in both P and IoU, with values of 87.08% and 76.23%, respectively, while UisNet achieves the second-best R and F1 at 85.53% and 86.09%. ABCNet shows the lowest R, F1, and IoU, with values of 84.08%, 84.71%, and 73.48%, respectively, while UNetFormer records the lowest P at 85.18%.

**Table 1:** Quantitative results of urban village extraction on the Beijing dataset using six methods. All results are reported as percentages (%).

Method	*P*	*R*	*F*1	*IoU*
UisNet (2022)	86.74	87.96	87.35	77.53
UV-SAM (2024)	86.41	87.49	86.95	76.91
ABCNet (2021)	84.85	86.87	85.85	75.21
CMTFNet (2023)	85.74	85.59	85.66	74.92
UNetFormer (2022)	84.84	85.62	84.84	74.26
MEBANet	**89.28**	**88.47**	**88.87**	**79.97**

**Table 2:** Quantitative results of urban village extraction on the Xi’an dataset using six methods. All results are reported as percentages (%).

Method	*P*	*R*	*F*1	*IoU*
UisNet (2022)	90.63	89.39	90.01	81.83
UV-SAM (2024)	89.26	87.31	88.27	79.01
ABCNet (2021)	89.03	86.83	87.92	78.44
CMTFNet (2023)	90.04	88.17	89.09	80.33
UNetFormer (2022)	89.57	88.41	88.98	80.15
MEBANet	**92.78**	**89.89**	**91.31**	**84.01**

**Table 3:** Quantitative results of urban village extraction on the Shenzhen dataset using six methods. All results are reported as percentages (%).

Method	*P*	*R*	*F*1	*I* *o* *U*
UisNet (2022)	86.66	85.53	86.09	75.58
UV-SAM (2024)	85.96	84.97	85.46	74.61
ABCNet (2021)	85.35	84.08	84.71	73.48
CMTFNet (2023)	87.08	85.96	86.52	76.23
UNetFormer (2022)	85.18	85.96	85.57	74.78
MEBANet	**88.44**	**87.29**	**87.86**	**78.35**

**Fig 12:**
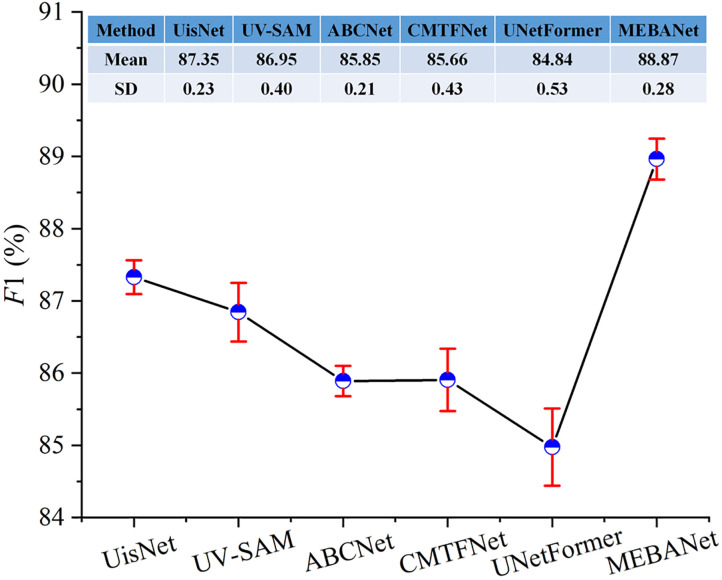
Mean and SD of F1 for different methods on the Beijing dataset.

**Fig 13:**
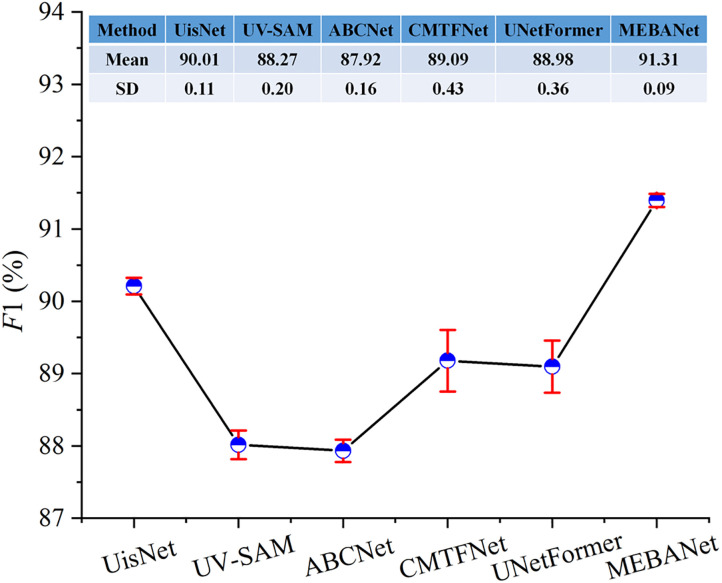
Mean and SD of F1 for different methods on the Xi’an dataset.

**Fig 14:**
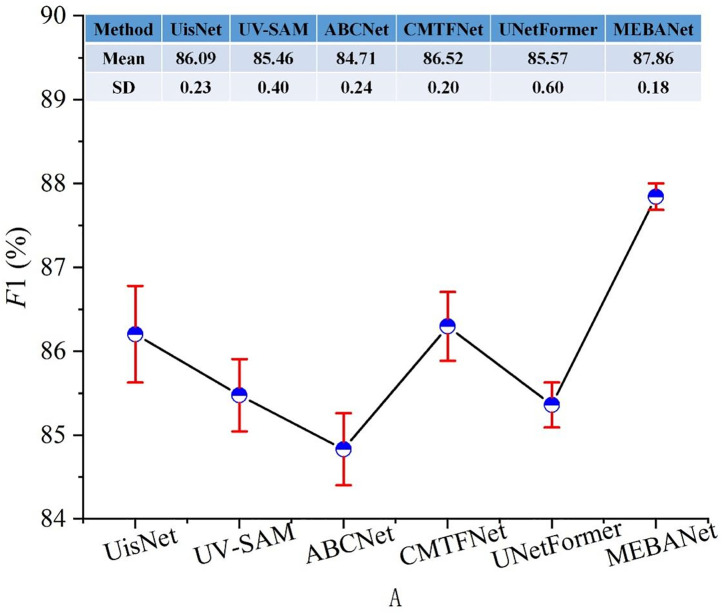
Mean and SD of F1 for different methods on the Shenzhen dataset.

The above results represent the average performance over five repeated experiments. Figs 12–14 present the mean and standard deviation (SD) of F1 for different methods on the Beijing, Xi’an, and Shenzhen datasets, respectively. It can be observed that MEBANet achieves the highest F1 across all datasets. Although it does not obtain the lowest SD on the Beijing dataset, where it ranks slightly behind UisNet and ABCNet, it still achieves the best overall accuracy. On the Xi’an and Shenzhen datasets, MEBANet demonstrates both the highest F1 and the most stable performance, highlighting its robustness and strong generalization capability. Combining the quantitative evaluation results and qualitative analysis, it is evident that MEBANet demonstrates the best performance in urban village extraction tasks. Across all three test datasets, MEBANet significantly outperforms the other compared methods.

## Discussion

### Ablation experiment

To validate the effectiveness of the constructed blocks, we conducted quantitative evaluation experiments on the Beijing dataset and Xi’an dataset, focusing on the performance contributions of three sub-modules: SFCB, MBAB, and DSB. Among these, MBAB and DSB are plug-and-play modules, while SFCB, as the core feature extraction structure, is an indispensable component of the model. Since traditional methods primarily focus on spatial domain feature extraction and often overlook the importance of frequency and channel domains, we specifically analyzed the contributions of FDE and CDE in detail. The ablation study results for MBAB and DSB are shown in Table 4, and the ablation results for the SFCB sub-module are presented in Table 5.

**Table 4:** Ablation study results of MBAB and DSB. All results are reported as percentages (%).

Method	Beijing dataset		Xi’an dataset	
	*F*1	*I* *_o_* *U*	*F*1	*I* *_o_* *U*
Backbone	87.38	77.58	88.59	79.52
Backbone+MBAB	88.15	78.81	90.04	81.89
Backbone+DSB	87.93	78.46	89.23	80.55
MEBANet	**88.87**	**79.97**	**91.31**	**84.01**

**Table 5:** Ablation study results of SFCB. All results are reported as percentages (%).

Method			Beijing dataset		Xi’an dataset	
SDE	FDE	CDE	*F*1	*I* *_o_* *U*	*F*1	*I* *_o_* *U*
√	—	—	87.24	77.36	88.78	79.82
—	√	—	87.07	77.09	88.28	79.02
—	—	√	86.22	75.77	87.31	77.48
√	√	—	88.49	79.35	90.49	82.63
√	√	√	**88.87**	**79.97**	**91.31**	**84.01**

From Table 4, it can be observed that on the Beijing dataset, using the MBAB alone increases the F1 by 0.77 percentage points (from 87.38% to 88.15%) and the IoU by 1.23 percentage points (from 77.58% to 78.81%). Using the DSB alone increases the F1 by 0.55 percentage points (from 87.38% to 87.93%) and the IoU by 0.88 percentage points (from 77.58% to 78.46%). When both MBAB and DSB are used together, the F1 increases by 1.49 percentage points (from 87.38% to 88.87%), and the IoU increases by 2.39 percentage points (from 77.58% to 79.97%).

On the Xi’an dataset, using the MBAB module alone increases the F1 by 1.45 percentage points (from 88.59% to 90.04%) and the IoU by 2.37 percentage points (from 79.52% to 81.89%). Using the DSB alone increases the F1 by 0.64 percentage points (from 88.59% to 89.23%) and the IoU by 1.03 percentage points (from 79.52% to 80.55%). When both MBAB and DSB are used together, the F1 increases by 2.72 percentage points (from 88.59% to 91.31%), and the IoU increases by 4.49 percentage points (from 79.52% to 84.01%). The above results demonstrate that both the MBAB and DSB play significant roles in improving model performance.

**Fig 15:**
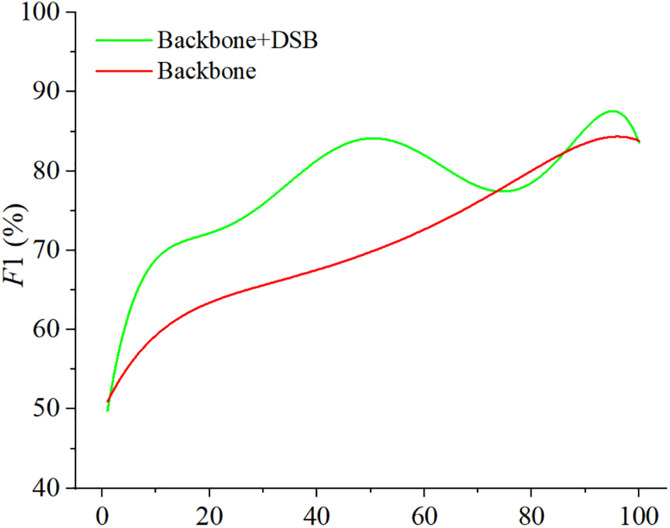
Comparison of training process with and without DSB.

Fig 15 illustrates the accuracy variations during the training process with and without DSB. The results demonstrate that while DSB introduces instability during training, it significantly accelerates the convergence of MEBANet. Moreover, the incorporation of DSB further improves the accuracy.

From the first three rows of Table 5, it can be observed that the accuracy is relatively low when each module is used independently. However, from the fourth and fifth rows, it becomes evident that the combined use of the modules enhances feature extraction capabilities, leading to improved accuracy in urban village extraction. Specifically, on the Beijing dataset, as indicated by the comparison between the first and fourth rows, the use of FDE results in an increase of 1.25 percentage points in F1 (from 87.24% to 88.49%) and 1.99 percentage points in IoU (from 77.36% to 79.35%). As shown in the comparison between the second and third rows, the use of SDE increases F1 by 1.42 percentage points (from 87.07% to 88.49%) and IoU by 2.26 percentage points (from 77.09% to 79.35%). Further, the inclusion of CDE, as seen in the fourth and fifth rows, results in a further improvement of 0.38 percentage points in F1 (from 88.49% to 88.87%) and 0.62 percentage points in IoU (from 79.35% to 79.97%).

On the Xi’an dataset, the use of FDE, as shown in the comparison between the first and fourth rows, increases F1 by 1.71 percentage points (from 88.78% to 90.49%) and IoU by 2.81 percentage points (from 79.82% to 82.63%). The comparison between the second and fourth rows indicates that SDE results in an increase of 2.21 percentage points in F1 (from 88.28% to 90.49%) and 3.61 percentage points in IoU (from 79.02% to 82.63%). Finally, as shown in the fourth and fifth rows, the incorporation of CDE leads to a further increase of 0.82 percentage points in F1 (from 90.49% to 91.31%) and 1.38 percentage points in IoU (from 82.63% to 84.01%).

## Backbone effectiveness analysis

To validate the effectiveness of the proposed feature extractor, SFCB, we conducted comparative experiments on the Beijing dataset and Xi’an dataset, comparing SFCB with several popular feature extractors. These include Transformer-based models such as Vision Transformer [33], Swin Transformer [47], and Mix-Transformer [32], as well as CNN-based models such as DenseNet [48], ResNet50 [49], and Xception [50]. The experimental results are shown in Table 6. Specifically, on the Beijing dataset, among the Transformer-based feature extractors, Vision Transformer performs the worst, but its F1 (87.55%) and IoU (77.86%) are still higher than those of the best-performing CNN-based feature extractor, ResNet50 (F1: 86.98%, IoU: 76.96%), by 0.57 percentage points and 0.90 percentage points, respectively. On the Xi’an dataset, among the Transformer-based feature extractors, Mix-Transformer performs the worst, but its F1 (88.77%) and IoU (79.81%) are still higher than those of the best-performing CNN-based feature extractor, DenseNet (F1: 88.36%, IoU: 79.15%), by 0.69 percentage points and 0.66 percentage points, respectively.

**Table 6:** Comparison results with different backbones. All results are reported as percentages (%).

Method	Beijing dataset		Xi’an dataset	
	*F*1	*I* *_o_* *U*	*F*1	*I* *_o_* *U*
Vision Transformer	87.55	77.86	89.29	80.65
Swin Transformer	87.58	77.90	89.16	80.44
Mix-Transformer	88.26	78.99	88.77	79.81
DenseNet	86.84	76.74	88.36	79.15
ResNet50	86.98	76.96	88.08	78.71
Xception	85.38	74.49	87.59	77.92
SFCB	**88.87**	**79.97**	**91.31**	**84.01**

Overall, SFCB significantly outperforms the other compared models on both datasets, validating its superiority in feature extraction. At the same time, Transformer-based feature extractors generally outperform CNN-based feature extractors, likely because Transformers are better at capturing global contextual information, thereby demonstrating stronger feature extraction capabilities in complex scenarios.

To further investigate the impact of the number of SFCB on urban village extraction task, we compared model performance when using 3, 4, and 5 SFCB. The experimental results are shown in Table 7, which demonstrates that the model achieves optimal performance with 4 SFCB on both datasets. Specifically, on the Beijing dataset, using 4 SFCB result in an F1 of 88.87% and an IoU of 79.97%, representing improvements of 1.32 percentage points and 2.11 percentage points, respectively, compared to using 3 SFCB, and improvements of 0.86 percentage points and 1.38 percentage points, respectively, compared to using 5 SFCB. On the Xi’an dataset, using 4 SFCB result in an F1 of 91.31% and an IoU of 84.01%, representing improvements of 1.68 percentage points and 2.79 percentage points, respectively, compared to using 3 SFCB, and improvements of 1 percentage point and 1.67 percentage points, respectively, compared to using 5 SFCB.

**Table 7:** Comparison results with different numbers of SFCB. All results are reported as percentages (%).

Method	Beijing dataset		Xi’an dataset	
	*F*1	*I* *_o_* *U*	*F*1	*I* *_o_* *U*
3	87.55	77.86	89.63	81.22
4	**88.87**	**79.97**	**91.31**	**84.01**
5	88.01	78.59	90.31	82.34

## Model transferability analysis

To investigate the robustness and transferability of MEBANet, we trained the model using the Beijing dataset and validated it on the Xi’an dataset. The experimental results are shown in Table 8. Due to differences in architectural styles between the two cities, the accuracy of all models decreased compared to the results in Table 1. Nevertheless, MEBANet still demonstrated the best performance, achieving P, R, F1, and IoU of 77.74%, 73.82%, 75.73%, and 60.94%, respectively. The second-best performance was achieved by UisNet, with P, R, F1, and IoU of 75.55%, 70.71%, 73.05%, and 57.54%, respectively, which are 2.19, 3.11, 2.68, and 3.40 percentage points lower than those of MEBANet. The worst performance was observed with ABCNet, which achieved P, F1, and IoU of 69.05%, 69.81%, and 53.63%, respectively, representing decreases of 8.69, 5.92, and 7.31 percentage points compared to MEBANet. Additionally, UV-SAM had the lowest R of 67.29%, which is 6.53 percentage points lower than that of MEBANet.

**Table 8:** Quantitative evaluation results of model transferability. All results are reported as percentages (%).

Method	Beijing dataset→ Xi’an dataset			
	*P*	*R*	*F*1	*I* *_o_* *U*
UisNet	75.55	70.71	73.05	57.54
UV-SAM	73.88	67.29	70.43	54.36
ABCNet	69.05	70.59	69.81	53.63
CMTFNet	74.58	70.69	72.58	56.96
UNetFormer	70.29	72.06	71.16	55.24
MEBANet	**77.74**	**73.82**	**75.73**	**60.94**

**Fig 16:**
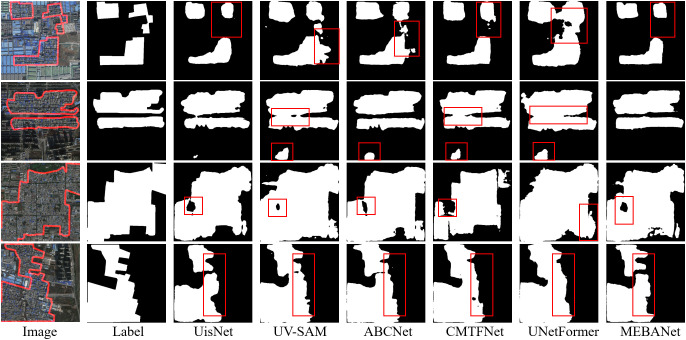
Visual results of model transferability.

Fig 16 demonstrates the visual results, and a comparison with Fig 9 also reveals that training and validation on different datasets lead to more positives, false negatives, irregular boundaries, and internal holes across all methods, including the proposed MEBANet. However, it can be observed that among all the methods, MEBANet performs the best, particularly in the second row.

The figure was produced by the authors of this study using the publicly available dataset from [22].

### Parameter fine-tuning

**Fig 17:**
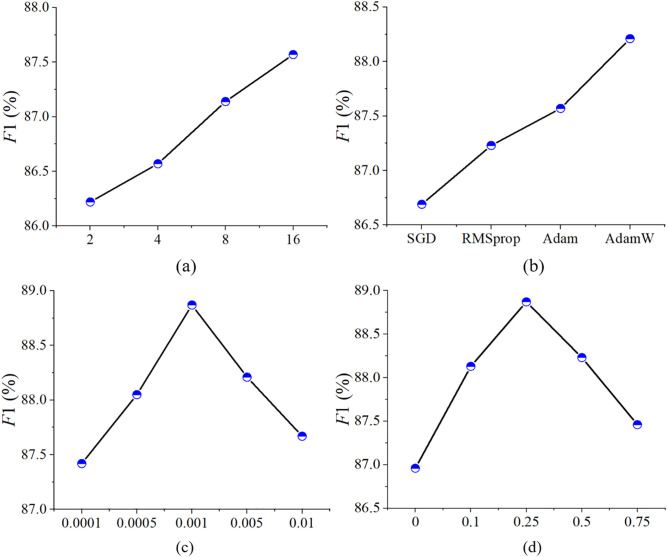
Parameter fine-tuning results: (a) batch size; (b) optimizer;(c) learning rate;(d) β.

The selection of appropriate model parameters is essential for achieving optimal performance. In this study, manual parameter tuning was employed to determine the optimal values for batch size, optimizer, learning rate, and β The tuning process was carried out in two stages: first, the parameters directly related to model training, namely batch size, optimizer, and learning rate, were determined; then, the model structure parameter, β, was adjusted. Initially, a learning rate of 0.005, the Adam optimizer, and β set to 0.25 were selected. The results, as shown in Fig 17(a), indicate that, through experimentation, the optimal batch size was identified as 16, which also represents the maximum feasible batch size due to hardware limitations. Subsequently, with the batch size fixed at 16 and the learning rate and β held constant, the optimizer was varied. The results, shown in Fig 17(b), demonstrate that AdamW was the most effective optimizer. Following this, with the optimizer fixed, the learning rate was adjusted, and the best performance was achieved at a learning rate of 0.001, as shown in Fig 17(c). Finally, with the first three parameters fixed, β was fine-tuned, and the results presented in Fig 17(d) reveal that a β value of 0.25 yielded the optimal performance.

### Model complexities

Table 9 presents the computational complexity and efficiency of different models. It can be seen that MEBANet is at an average level in terms of model efficiency. MEBANet has 26.71M parameters, which is slightly higher than the lightweight models ABCNet and UNetFormer, much lower than UV-SAM, and at a similar level to UisNet and CMTFNet. In terms of computational complexity, MEBANet is significantly lower than UisNet and UV-SAM, while maintaining comparable efficiency to CMTFNet. Notably, MEBANet’s inference time reaches 20.54ms, which is only slightly slower than ABCNet and UNetFormer, but shows a significant advantage over UisNet, CMTFNet, and UV-SAM.

**Table 9:** Computational complexity and efficiency of different methods.

Method	Params (M)	FLOPs (Gbps)	Inference time (ms)
ABCNet (2021)	13.81	15.31	15.44
UisNet (2022)	28.12	69.22	86.43
UNetFormer (2022)	11.72	11.70	15.59
CMTFNet (2023)	30.07	32.84	40.71
UV-SAM (2024)	316.20	76.58	96.45
MEBANet	**26.71**	**34.78**	20.54

### Limitations and future work

Although the proposed MEBANet achieves high accuracy in urban village extraction task and its effectiveness has been validated through experiments, there are still several areas worthy of further investigation:

1) Dataset expansion: This study was tested only on datasets from three cities in China. While the results are promising, the scope of the research is currently limited. To enhance the generalizability of the model, future work will involve testing it on datasets from additional regions, including cities with different architectural styles and urban features, such as those from Africa and South America. This will help validate the model’s adaptability to diverse geographical and cultural contexts.
2) Utilization of multi-modal data: This study relies solely on optical remote sensing images for urban village extraction. Considering that multi-modal data provide additional perspectives, future research will explore the integration of multi-modal data, such as building height data and SAR imagery, to further enhance the model’s recognition accuracy and robustness.
3) Transfer learning issue: Although MEBANet has been tested with transfer learning and shows better performance compared to others methods, the issue of significant accuracy degradation on unseen data remains unresolved. In future research, we will address this limitation by exploring domain adaptation and self-supervised learning techniques to enhance the model’s robustness and ensure consistent performance across diverse datasets.

## Conclusions

This study addresses the complexity and challenges of urban village extraction by proposing an innovative method named MEBANet. The proposed MEBANet significantly enhances the accuracy and robustness of urban village extraction through the synergistic integration of three core modules: the SFCB, the MBAB, and the DSB. Experimental results demonstrate that MEBANet consistently outperforms existing methods on the Beijing, Xi’an and Shenzhen datasets in terms of overall performance. Cross-dataset transfer experiments further validate its strong generalization capability. Ablation studies confirm the individual contributions and effectiveness of each module. Future research directions include testing the accuracy of urban village extraction on different regions, incorporating multi-modal data into the task to improve accuracy, and addressing the challenge of generalization to unseen data.
